# Ethical leadership and followers’ career satisfaction, mobility, and promotability: A P-E fit perspective

**DOI:** 10.3389/fpsyg.2022.927146

**Published:** 2022-11-02

**Authors:** Ruobing Xi, Kun Yu, Yao Ge, Peiyue Cao

**Affiliations:** ^1^Antai College of Economics and Management, Shanghai Jiao Tong University, Shanghai, China; ^2^School of Labor and Human Resources, Renmin University of China, Beijing, China

**Keywords:** ethical leadership, person-environment fit, career success, mobility, promotability, career satisfaction

## Abstract

The purpose of this paper is to examine the effect of ethical leadership on followers’ subjective and objective career success from a P-E fit perspective. Specifically, the mediating effects of demands-abilities fit, needs-supplies fit, and person-organization fit in the relationship between ethical leadership and employee subjective (i.e., career satisfaction) and objective career success (i.e., mobility and promotability) were investigated. We collected two-wave data from 160 employees and used hierarchical regressions to test the hypotheses. The findings revealed that ethical leadership had a positive effect on employee career satisfaction, mobility, and promotability. Moreover, employee demands-abilities fit mediated the relationship between ethical leadership and career mobility and promotability; needs-supplies fit and person-organization fit mediated the relationship between ethical leadership and employee career satisfaction. Theoretical and empirical implications were discussed.

## Introduction

In this era of boundaryless careers, successful employees remain value-added to their present employer and are viewed as marketable by other organizations (Eby et al., 2003). Employees’ career success, which could be both subjectively and objectively defined (Judge et al., 1995), not only represents “the positive psychological and work-related outcomes accumulated as a result of one’s work experience” (Seibert and Kraimer, 2001), but also reflects their essential contributions to organizational goal achievement (van Dam, 2004; Ng et al., 2005). Subjective career success, measured in terms of career satisfaction, focuses on individual subjective appraisals of their own career-related achievements (Seibert and Kraimer, 2001); while objectively career success focuses on some observable exoteric metrics, including salary, promotability, and mobility (Arthur and Rousseau, 1996). Previous literature has delved into the predictors of objective and subjective career success. Among predictors, examination of the relevant literature supported the link between leadership, which plays a crucial role in the construction of followers’ work experience (Demirtas and Akdogan, 2015), and subordinates’ career success (e.g., Riaz and Haider, 2010; Kim and Beehr, 2018; Wang et al., 2019). Effective leaders offer valuable sponsorship and support to enhance their subordinates’ careers, such as training and skill development opportunities, work-related resources, and adequate motivation (Ng et al., 2005). Especially, as recent ethics scandals in business and government highlight the importance of virtue and morality, the effects of ethical leadership style (Brown et al., 2005; Demirtas and Akdogan, 2015) on subordinates’ career success (Alioullar and Karabey, 2018; Dust et al., 2018) has attracted increased attention.

However, these studies have exclusively focused on the effects of ethical leadership on subjective career success, to the large neglect of objective career success. In fact, subjective career success that captures individuals’ self-defined career goal achievement is not sufficient to reflect their competitiveness in the current organization and the whole labor market. Hence, it is of great importance to consider objective career success when exploring the relationship between ethical leadership and career success.

**Figure 1:**
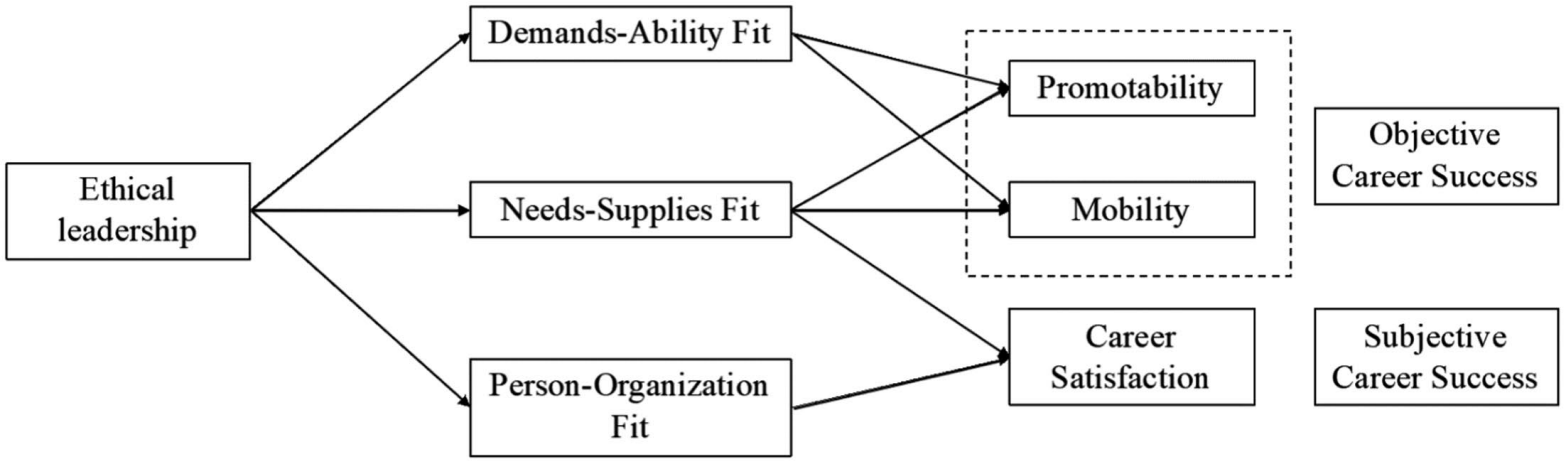
Hypothesized research model.

Therefore, the present study aims to investigate how ethical leadership exerts an impact on both objective and subjective career success based on the person-environment fit theory (Cable and Edwards, 2004). Traditionally, social exchange theory and social learning theory have served as the most common theoretical basis in ethical leadership literature (Brown et al., 2005), which emphasize norms of reciprocity and role modeling. However, career success is shaped by the complicated effects of psychological, social, and organizational factors (Arthur, 2014; Tams and Arthur, 2010), and cannot be simply explained by perceived obligation and observation. In contrast, P-E fit is defined as the degree of congruence or match between a person and the environment (Schneider, 1987; Pervin, 1989). It suggests that when individuals acquire abilities and skills that meet the job requirements, obtain resources that meet their needs, and experience value congruence with their organizations, they are more likely to generate positive attitudes, behaviors, and performance (Cable and DeRue, 2002; Cable and Edwards, 2004; Kristof-Brown et al., 2005). Therefore, the P-E fit theory provides essential theoretical support for explaining how ethical leaders stimulate subordinates’ career success by improving their fit with jobs and organizations. Derived from the P-E fit theory, we propose that demands-ability fit, needs-supplies fit, and person-organization fit mediate the positive relationship between ethical leadership and both subjective and objective career success. Figure 1 displays the hypothesized model of the present study.

By addressing the above issues, the current research contributes to the literature in mainly three ways. First, investigating the relationship between ethical leadership and career success from the P-E fit perspective sheds some new light on uncovering the dynamic management process in which ethical leaders foster subordinates’ career success. Second, connecting ethical leadership and subordinates’ objective career success helps to fully reflect the explanatory power of ethical leadership on subordinates’ competitiveness in the current organization and the whole labor market. Third, the introduction of multiple fit dimensions as parallel mechanisms also contributes to better distinguishing the different effects of ethical leadership on subjective and objective career success.

## Hypotheses development

### The mediating role of demands-abilities fit

Based on the P-E fit theory, the concept of P-E fit is defined as the degree of congruence or match between a person and the environment, which results in different attitudes, behaviors, and performances (Schneider, 1987; Pervin, 1989). On the one hand, the P-E fit paradigm comprises two traditions, complementary fit and supplementary fit (Muchinsky and Monahan, 1987). On the other hand, the P-E fit comprises three dimensions, namely demands-abilities fit, needs-supplies fit, and person-organization fit.

Demands-abilities fit illustrates the congruence between one’s capabilities and the job requirements (Cable and Edwards, 2004). If employees fully utilize the opportunities to diversify and update their portfolio of skills, they will gain a higher level of demands-abilities fit and get qualified for principal positions. Current fit literature has generally accepted that the congruence between individuals’ abilities and job demands could predict higher job performance (Caldwell and O’Reilly, 1990; Edwards, 1991; Kristof-Brown et al., 2005), which would increase employees’ perception of internal and external employability.

Ethical leadership is “the demonstration of normatively appropriate conduct through personal actions and interpersonal relationships, and the promotion of such conduct to followers through two-way communication, reinforcement, and decision-making.” (Brown et al., 2005). According to the conceptualization of ethical leadership, ethical leaders would show individualized consideration to their subordinates, including coaching, counseling, and paying particular attention to their development, growth, and success (Chi and Pan, 2012). Such behaviors inspire subordinates to improve their capabilities and increase the level of demands-abilities fit. Second, ethical leaders exert idealized influence on their subordinates and serve as role models that demonstrate and communicate their high ethical values and goal expectations, using rewards and punishments to encourage subordinates to meet such requirements (Bedi et al., 2016). Hence, subordinates are more likely to learn (Ali et al., 2021) and acquire more diverse and advanced skills and become more competent and fit for current and even higher-level jobs. To sum up, we propose that ethical leadership would facilitate employees’ objective career success, such as a high level of mobility and promotability (Arthur and Rousseau, 1996), by enhancing demands-abilities fit. Thus, we predict the following:

*H1*: Demands-abilities fit mediated the relationship between ethical leadership and subordinates’ objective career success.

### The mediating role of needs-supplies fit

According to the P-E fit theory, needs-supplies fit captures the extent of match between employees’ needs and the supplies of their jobs, such as resources, achievement, salaries, interpersonal relationships, and meaningful work (Edwards, 1991). Individuals possess diverse needs, namely autonomy, affiliation, social support, self-actualization, and so forth. Theories of need fulfillment (Locke, 1976) propose that when individuals’ needs are satisfied, they generate more positive work attitudes and behaviors. Basic psychological needs fulfillment acts as a wide-used example in the N-S fit domain (Mayer et al., 2008), which is defined as the innate and universal psychological nutriments that contain the need for autonomy, the need for competence, and the need for relatedness (Ryan and Deci, 2000). Satisfaction of basic psychological needs is deemed necessary for optimal and ongoing psychological development, integrity, and well-being (Ryan and Deci, 2000), such as job satisfaction (Mayer et al., 2008) and work engagement (Deci et al., 2001).

As leaders are deemed an integral part of subordinates’ organizational context, they play a pivotal role in providing necessary conditions to satisfy subordinates’ needs and derive the commensurate supply (Caplan, 1987; Chiniara and Bentein, 2016). Moreover, the basic psychological needs fulfillment perspective suggests several ways in which ethical leadership fosters subordinates’ N-S fit. First, ethical leaders provide subordinates opportunities to express themselves in decision-making and take their ideas and concerns into consideration (Brown et al., 2005; Den Hoogh and Den Hartog, 2008; Piccolo et al., 2010). Consequently, subordinates experience a high level of autonomy and influence over work-related decisions, and their needs for autonomy are fulfilled. Second, since ethical leadership highlights individualized consideration and expresses care and concern to their subordinates, such leaders provide employees with opportunities to enhance or update their skills and assist them to pursue their goals. Hence, they satisfy followers’ needs for competence. Finally, as a moral person, an ethical leader exhibits appropriate behaviors in a consistent, fair, trustworthy manner (Brown and Treviño, 2006). A moral manager, such as a leader cares for followers, makes fair and principled decisions, and addresses followers’ needs for safety and security (Demirtas and Akdogan, 2015). Therefore, ethical leaders engender relational attachments in organizations that meet followers’ needs for relatedness. To sum up, ethical leaders fulfill subordinates’ basic psychological needs and are positively related to their needs-supplies fit.

Previous empirical literature and meta-analysis revealed that N-S fit is conducive to career satisfaction because employees judge the success of their careers primarily on whether their job has enabled them to satisfy their needs (Cable and DeRue, 2002). When employees perceive that their jobs fit their financial, social, or psychological needs, they tend to become more satisfied with and committed to their jobs, and, thereby, invest more time and energy in their work and set higher performance standards (Chi and Pan, 2012), resulting in higher performance and higher perceived employability. Based on the above arguments, we propose the following hypothesis:

*H2*: Needs-supplies fit mediates the positive relationship between ethical leadership and subordinates” subjective career success.

*H3*: Needs-supplies fit mediates the positive relationship between ethical leadership and subordinates’ objective career success.

### The mediating role of person-organization fit

Based on the P-E fit theory, person-organization fit captures the compatibility between the commensurate individual and organizational characteristics, among which value congruence acts as the most widely accepted defining operationalization (Kristof, 1996; Verquer et al., 2003). Theoretically, value congruence has a pivotal impact on employees’ positive attitudes because individuals are more trusting of and attracted to those who possess similar values (Cable and Edwards, 2004). Employees with similar values share similar cognitive processes and means of communication. Thus, they enjoy improved communications and increased predictability in social interactions, resulting in closer interpersonal relationships and a higher level of satisfaction (Bluedorn et al., 1999; Schminke et al., 2005). Empirically, several meta-analyses indicated that P-O fit or value congruence was a direct and significant predictor of employee satisfaction, commitment, and intention to remain with an organization (Verquer et al., 2003; Cable and Edwards, 2004; Kristof-Brown et al., 2005; Hoffman and Woehr, 2006); while the links between P-O fit and behavioral outcomes were rather weak, such as job performance and overall performance (Kristof-Brown et al., 2005). Hence, P-O fit may significantly impact individuals’ career satisfaction rather than mobility and promotability.

Ethical leaders are considered to shape perceptions of ethical climate and culture in the organization, which engenders subordinates’ internalization of ethical values and attitudes (Brown and Treviño, 2006). As an attractive role models, ethical leaders act as a crucial source of ethical guidance for organization members (Brown et al., 2005). Leaders set ethical standards and communicate them to subordinates, using rewards and punishments to hold subordinates accountable for ethical conduct (Treviño et al., 2003). Such an ethical climate motivates subordinates to discuss and internalize the meaningfulness of promoting ethical values in the workplace (Mayer et al., 2012; Walumbwa et al., 2012; Huang and Paterson, 2017). Under the supervision of ethical leaders, subordinates form an identification with ethical norms and values that are encouraged by the whole organization, such as knowledge sharing (Men et al., 2020; Anser et al., 2021) and voicing (Lee et al., 2017). Thus, they are more likely to experience a high level of person-organization fit. The mediating effect of person-organization fit in the ethical leadership-career satisfaction relationship is hypothesized as follows:

*H4*: Person-organization fit mediates the positive relationship between ethical leadership and subordinates’ subjective career success.

## Materials and methods

### Participants and procedures

Participants for this study were recruited from employees in an electronic commerce company in China. The electronic commerce industry has been growing rapidly in China in recent years, in which promotions are more frequent and employee mobility between companies is more common. This makes employees in this industry an ideal sample for our research. Two wave survey data were collected with a one-month time interval. In the first wave, participants completed a questionnaire assessing their immediate supervisor’s ethical leadership and various demographic variables. One month later, we sent the participants another questionnaire measuring their demands-ability fit, needs-supplies fit, person-organization fit, subjective career success (career satisfaction), and objective career success (mobility and promotability). Ethical aspects, informed consent, and confidentiality were mentioned at the beginning of the questionnaire, and permission was obtained from the organization.

Overall, 171 employees participated in the first-wave survey, and 163 employees completed it with a useful response rate of 95.32 percent. Then, we sent questionnaires to these 163 employees in the second-wave survey; a total of 160 employees completed both questionnaires with usable information for a total usable response rate of 93.57 percent for the two waves. Regarding the sample characteristics, 55.62 percent of the participants were female and 44.38 percent were male. The mean age of the whole sample was 29.26 years (SD = 5.33), and the length of organizational tenure was 5.37 years (SD = 4.8). In terms of education, 40.6 percent of the participants finished college, 49.4 percent received a Bachelor’s degree, and 10% acquired a Master’s degree.

### Measures

To avoid conceptual inconsistency, this study followed the translation-back translation procedure recommended by Brislin (1980). First, three bilingual researchers translated the questionnaires from English into Chinese. Second, the questionnaires were back-translated into English.

#### Ethical leadership

Ethical leadership was measured with a 10-item Ethical Leadership Scale developed by Brown et al. (2005). Participants rated their direct leaders on a 7-point Likert scale ranging from 1 (strongly disagree) to 7 (strongly agree). One sample item is “My leader conducts his/her personal life in an ethical manner.” The Cronbach’s α of this scale is 0.95 in this study, while in other studies Cronbach’s α was 0.92 (Ali et al., 2021), 0.93 (Abdullah et al., 2019), 0.88 (Khan et al., 2019), and 0.92 (Huang and Paterson, 2017).

#### Demands-abilities fit

Demands-abilities fit was measured with three items developed by Cable and DeRue (2002). Responses could range from 1 (strongly disagree) to 7 (strongly agree), and a higher score indicated a higher level of fit. One sample item is “My personal abilities and education provide a good match with the demands that my job places on me.” The Cronbach’s α of this scale is 0.85 in this study, while in other studies Cronbach’s α was 0.89 (Tims et al., 2016) and 0.88 (Gabriel et al., 2014).

#### Needs-supplies fit

We measured needs-supplies fit by using Cable and DeRue’s (2002) 3-item scale. Participants were asked to rate on a 7-point Likert scale ranging from 1 (strongly disagree) to 7 (strongly agree). One sample item is “There is a good fit between what my job offers me and what I am looking for in a job.” The reliability of the scale in this study was α = 0.83, while in other studies Cronbach’s α was 0.90 (Gabriel et al., 2014) and 0.89 (Rehfuss et al., 2012).

#### Person-organization fit

Person-organization fit was assessed by three items developed by Cable and DeRue (2002). Participants rated on a 7-point Likert scale ranging from 1 (strongly disagree) to 7 (strongly agree). One sample item is “The things that I value in life are very similar to the things that my organization values.” The reliability of the scale in this study was α = 0.90, while in other studies Cronbach’s α was 0.92 (Gabriel et al., 2014), and 0.89 (Rehfuss et al., 2012).

#### Subjective career success

Career satisfaction was assessed with the 5-item scale formulated by Greenhaus et al. (1990). A 7-point Likert scale ranging from 1 (strongly disagree) to 7 (strongly agree) was used. One sample item is “I am satisfied with the progress I have made toward meeting my overall career goals.” The Cronbach’s α of this scale is 0.93 in this study.

#### Objective career success

Promotability was measured with a 3-item scale created by De Pater et al. (2009). Respondents were asked to indicate “To what extent do you have the capabilities to successfully perform in higher-level jobs” and the other two items were based on a 7-point Likert scale ranging from 1 (strongly disagree) to 7 (strongly agree). The Cronbach’s α of the scale in this study was α = 0.82.

We assessed external mobility in the broader labor market using a three-item scale. Two items were taken from Tepper (2000; i.e., “If I were to quit my job, I could find another job that is just as good,” and “I would have no problem finding an acceptable job if I quit.”) to assess mobility between jobs. One item (i.e., “I could easily find a decent job with my abilities and experiences”) was added to capture perceived mobility in a more general way. Each item was rated on a 7-point Likert scale ranging from 1 (strongly disagree) to 7 (strongly agree). To test the psychometric characteristics of the three-item structure, we performed exploratory factor analysis (EFA) with principal component analysis extraction and varimax rotation, which yielded a 1-factor solution that accounted for 72.86% of the variance. The Cronbach’s α of the three-item scale was.81, which was also better than Tepper’s (2000) original two-item scale (i.e., Cronbach’s α = 0.66).

#### Control variables

Based on the meta-analyses of career success antecedents by Ng et al. (2005), age, gender, and education level were proven to be related to perceptions of career success. Therefore, we controlled these variables in all subsequent analyses.

## Results

### Preliminary analyses

Harman’s single-factor analysis was performed to detect the common method bias (CMB). The results showed that four factors with an eigenvalue larger than 1 explained 70.80% of the variance. The first factor explained 46.87% of the variance, which was below the recommended cutoff value of 50% (Harrison et al., 1996), indicating that CMV was not a serious concern for the current research. We also conducted a series of confirmatory factor analyses (CFA) to examine the construct validity of measurements reported by participants. Table 1 presents the results of the tests of competing measurement models conducted by Mplus 7. Model 1 contains six factors for the concept of ethical leadership, demands-abilities fit, needs-supplies fit, person-organization fit, subjective career success, and objective career success. To reduce the model size, two parcels were created for ethical leadership. We combined the item with the highest loading and lowest loading into a new item repeatedly (Little et al., 2002). Fit indices showed that model 1 has the best goodness of fit: *χ*^2^ =495.59, df = 194; comparative fit index (CFI) = 0.95; Tucker–Lewis index (TLI) = 0.94; root mean square error of approximation (RMSEA) = 0.07; standardized root mean square residual (SRMR) = 0.06.

**Table 1:** Results of confirmatory factor analysis.

Model	*χ*^2^ (df)	*Δχ*^2^ (*Δdf*)	CFI	TLI	RMSEA	SRMR
Model 1	495.59^***^(194)		0.95	0.94	0.07	0.06
Model 2	934.53^***^(208)	438.94(14)	0.76	0.73	0.15	0.09
Model 3	1180.02^***^(209)	684.43(15)	0.68	0.65	0.17	0.10

CFI, comparative fit index; TLI, Tucker–Lewis index; RMSEA, root mean square error of approximation. Model 1: Ethical leadership, demands-abilities fit, needs-supplies fit, person-organization fit, subjective career success, and objective career success. Model 2: Ethical leadership, demands-abilities fit + needs-supplies fit + person-organization fit + subjective career success + objective career success. Model 3: Ethical leadership + demands-abilities fit + needs-supplies fit + person-organization fit + subjective career success + objective career success.

Compared with the six-factor model 1, a two-factor model combining demands-abilities fit, needs-supplies fit, person-organizations fit, subjective career success and objective career success did not fit the data well: *χ*^2^ = 934.53, df = 208; CFI = 0.76; TLI = 0.73; RMSEA = 0.15; SRMR = 0.09. Neither did the single-factor model, which had all the indicators of ethical leadership, person-environment fit, and career success loading on a single construct: *χ*^2^ = 1180.02, df = 209; CFI = 0.68; TLI = 0.65; RMSEA = 0.17; SRMR = 0.10. All these CFA results of comparative models illustrate that Model 1 with six distinguished factors had significant discriminant validity.

### Descriptive analysis

Table 2 presents means, standard deviations, and correlations among the variables, while Cronbach’s alpha for each scale variable is reported along the diagonal. Ethical leadership is positive related with demands-abilities fit (*r* = 0.402, *p* < 0.01), needs-supplies fit (*r* = 0.478, *p* < 0.01), person-organization fit (*r* = 0.514, *p* < 0.01), career satisfaction (*r* = 0.490, *p* < 0.01), promotability (*r* = 0.323, *p* < 0.01), mobility (*r* = 0.438, *p* < 0.01). Regarding the correlations between ethical leadership and mediation variables and dependent variables, all three types of fit are significantly related to both subjective career success and objective career success.

**Table 2:** Descriptive statistics among research variables.

	Mean	SD	1	2	3	4	5	6	7	8	9	10
1. Age	29.27	5.33										
2. Gender	1.56	0.50	0.003									
3. Education	3.69	0.64	0.224^**^	−0.014								
4. Ethical leadership	5.34	1.30	−0.089	−0.049	−0.126	*(0.95)*						
5. Demands-abilities fit	5.04	1.26	0.021	−0.043	−0.088	0.402^**^	*(0.85)*					
6. Needs-supplies fit	4.51	1.35	0.031	−0.055	−0.155	0.478^**^	0.623^**^	*(0.83)*				
7. Person-organization fit	4.77	1.34	0.013	−0.084	−0.107	0.514^**^	0.668^**^	^.^775^**^	*(0.90)*			
8. Career satisfaction	4.80	1.42	0.019	−0.075	−0.108	0.490^**^	0.768^**^	0.842^**^	0.599^**^	*(0.93)*		
9. Promotability	5.08	1.20	0.027	−0.061	−0.062	0.323^**^	0.527^**^	0.515^**^	0.552^**^	0.466^**^	*(0.82)*	
10. Mobility	4.81	1.17	0.006	−0.155	−0.058	0.438^**^	0.603^**^	0.529^**^	0.723^**^	0.514^**^	0.648^**^	*(0.81)*

*N* = 160. Cronbach’s *α* are reported in parentheses along the diagonal.

** *p* < 0.01.

### Hypothesis testing

Regression analyses using Mplus 7 (Muthén and Muthén, 1998–2012) were performed to test study hypotheses. To provide multivariate tests of the study hypotheses, we performed a series of regression analyses. In all the regression models, age, gender, and educational level were controlled. To minimize the multicollinearity effects, we employed the mean-centered approaches on all variables. Table 3 presents the results of these analyses. Model 1, 2, and 3 indicated that ethical leadership had a significantly positive impact on demands-abilities fit (*β*_1_ = 0.379, *p* < 0.01), needs-supplies fit (*β*_2_ = 0.486, *p* < 0.01), and person-organization fit (*β*_3_ = 0.524, *p* < 0.01).

**Table 3:** Results of regression analysis.

	D-A Fit	N-S Fit	P-O Fit	Career Satisfaction		Promotability		Mobility	
	Model 3	Model 2	Model 1	Model 4–1	Model 4–2	Model 5–1	Model 5–2	Model 6–1	Model 6–2
Intercept	3.02	2.23^*^	2.14^*^	2.13	0.037	3.28	1.38	3.23	1.62
Age	0.016	0.025	0.018	0.020	−0.001	0.010	−0.0003	0.014	0.002
Gender	−0.060	−0.092	−0.160	−0.149	−0.047	−0.314	−0.260	−0.110	−0.054
Education	−0.103	−0.248	−0.125	−0.145	0.049	0.030	0.040	−0.069	0.021
Ethical leadership	0.379^**^	0.486^**^	0.524^**^	0.530^**^	0.069	0.389^**^	0.118^*^	0.297^**^	0.027
D-A Fit					0.041		0.540^**^		0.316^**^
N-S Fit					0.633^**^		−0.015		0.158
P-O Fit					0.261^**^		0.141		0.140
*F*	7.84	12.76	14.62	12.95	63.83	10.43	29.21	4.83	12.39
*R* ^2^	0.168	0.248	0.274	0.251	0.746	0.212	0.574	0.111	0.363
*ΔR* ^2^					0.495^**^		0.362^**^		0.252^**^

*N* = 160.

* *p* < 0.05;

** *p* < 0.01.

*H1* predicts that demands-abilities fit mediates the relationship between ethical leadership and objective career success. Model 5–1 and 6–1 demonstrated that ethical leadership was positively associated with promotability (*β*_5–1_ = 0.389, *p* < 0.01) and mobility (*β*_6–1_ = 0.297, *p* < 0.01). When ethical leadership and demands-abilities fit were both included in the regression equation, as shown in Model 5–2 and 6–2, demands-abilities fit had a significant positive influence on promotability (*β*_5_ = 0.540, *p* < 0.01) and mobility (*β*_6_ = 0.316, *p* < 0.01). However, the influences of ethical leadership were considerably weakened: the effect on promotability was less significant and lesser in magnitude (*β*_5–2_ = 0.118, *p* < 0.05), whereas the effect on mobility became insignificant (*β*_6–2_ = 0.027). It suggested that demands-abilities fit fully mediates the positive relationship between ethical leadership and mobility, while partially mediates the relationship between ethical leadership and promotability. Thus, *H1* was preliminarily supported.

*H2* and *H4* assert that needs-supplies fit and person-organization fit mediate the relationship between ethical leadership and subjective career satisfaction. Model 4–1 indicated that ethical leadership is positively related to career satisfaction (*β*_4–1_ = 0.530, *p* < 0.01). As shown in model 4–2, needs-supplies fit and person-organization fit significantly impacted ethical leadership (*β_n−s fit_* = 0.633, *p* < 0.01; *β_p−o fit_* = 0.261, *p* < 0.01), whereas the effect of ethical leadership was not significant in this model. These results suggested that needs-supplies fit and person-organization fit fully mediate the positive relationship between ethical leadership and subjective career satisfaction. Thus, *H2* and *H4* were preliminarily supported.

*H3* suggests that needs-supplies fit mediates the relationship between ethical leadership and objective career success. As demonstrated in Model 5–2 and 6–2, needs-supplies fit did not have any significant impact on promotability or mobility (*β*_*N−S Fit* 5−2_ = −0.015, *p* = 0.844; *β*_*N−S Fit* 6−2_ = 0.158, *p* = 0.098). Such results did not support *H3*.

**Figure 2:**
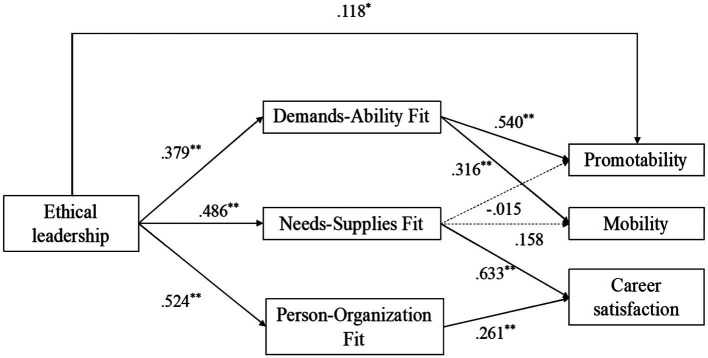
Results of hypotheses testing.

To provide further evidence regarding *H1*, *H2*, and *H4*, the path analytic approach was used to estimate the mediation effect (Preacher et al., 2007; Hayes, 2013). We used Model 4 of the PROCESS macro (Hayes, 2013) with a bootstrapping method to estimate the indirect effect. Figure 2 illustrates the indirect effect model. In the objective career success model, demands-abilities fit partially mediated the positive association between ethical leadership and career success promotability (indirect effect = 0.21, 95% CI = [0.10, 0.32]; direct effect = 0.12, 95% CI = [−0.01, 0.23]); it also fully mediated the relationship between ethical leadership and mobility (indirect effect = 0.12, 95% CI = [0.04, 0.23]; direct effect = 0.03, 95% CI = [−0.11, 0.17]). In the subjective career success model, supporting *H2b* and *H3b*, needs-supplies fit (indirect effect = 0.31, 95% CI = [0.20, 0.43]) and person-organization fit (indirect effect = 0.14, 95% CI = [0.03, 0.26]) both meditated the positive effect of ethical leadership on career satisfaction (direct effect = 0.07, 95% CI = [−0.04, 0.17]). These results provided further support for *H1*, *H2*, and *H4*.

## Discussion

The present research found that ethical leadership has a positive and significant impact on demands-abilities fit, needs-supplies fit, and person-organization fit, which, in turn, promote employees’ subjective (career satisfaction) and objective career success (mobility and promotability). Consistent with our hypotheses, demands-abilities fit mediated the relationship between ethical leadership and objective career success (i.e., mobility and promotability), while needs-supplies fit and person-organization fit both mediated the association between ethical leadership and subjective career success (i.e., career satisfaction). However, the needs-supplies fit did not mediate the relationship between ethical leadership and objective career success, neither mobility nor promotability. The main reason was that we did not find a significant association between needs-supplies fit and objective career success. This is probably because, although on one hand, the fulfillment of employees’ needs by supplies from the organization lets them work harder and gain higher performance and higher perceived employability (Chi and Pan, 2012), on the other hand, the satisfaction of needs also lead to employees’ tendency to stay in where they are (Schneider, 1987), which may hinder their potential promotion or mobility.

### Theoretical and practical implications

Theoretically, the current research extends the literature on ethical leadership and career success in three ways. First, the concept of P-E fit functions as a novel explanatory mechanism for the positive effect of ethical leadership on career success. Social exchange theory and social learning theory focalize on the *moral person* component of ethical leadership, indicating that employees behave ethically because leaders are honest, dedicated, and fair, cares genuinely about employees’ well-being, and address their development needs (Bedi et al., 2016). However, such an explanation neglects the moral manager component and blurs the main differences between ethical leadership and other leadership styles. Contrarily, the P-E fit theory highlights the match among individuals, leaders, organizations, and jobs (Pervin, 1989), providing essential theoretical support for the positive impact of the congruence between individual and organizational ethical climate, and demonstrates the whole process by which ethical leaders improve their subordinates’ P-E fit and thus enhance their competitiveness in the labor market. Second, by examining the role of ethical leadership on subordinates’ objective career success in addition to subjective career success, the current research draws a more complete picture of the explanatory power of ethical leadership on subordinates’ career success. Third, the introduction of demands-abilities fit, needs-supplies fit, and person-organization fit as parallel mechanisms also sheds light on better distinguishing the different effects of ethical leadership on subjective and objective career success.

This study also offers useful suggestions for management practitioners. First, the findings demonstrate that ethical leadership can be a useful way to facilitate subjective (career satisfaction) and objective career success (mobility and promotability) *via* demands-abilities fit, needs-supplies fit, and person-organization fit. Thus, we suggest leaders exhibit more ethical behaviors, engage in transparent, fair, honest, and trustworthy actions, and express care and concern for employees. Leaders are also encouraged to make fair and balanced decisions, set ethical standards, communicate them with employees, and use rewards and punishments to promote ethical conduct and create an ethical climate in organizations. In such an ethical organization, employees gain sufficient supply from leaders and enhance their needs-supplies fit. Employees are suggested to make full use of the resources given by leaders so that they can improve career-relevant skill portfolios and get a higher level of demands-abilities fit. Moreover, organizations and individuals are supposed to put the effort into increasing ethical value congruence. In this way, employees are motivated and empowered to achieve both subjective and objective career success.

### Limitations and future directions

We must acknowledge several limitations of our studies that imply directions for future research. First, although this study conducted a time-lagged survey, this is a cross-sectional study in essence. The relationship between ethical leadership and subjective or objective career success and the mediation effect of fit variables are only in a temporal order instead of a solid causal conclusion. A three-wave longitudinal design or experimental design is needed to validate this model and further investigate how these variables would shape each other over time. Second, we only consider the P-E fit theory as the explanation and mediation mechanism to link ethical leadership and subjective and objective career success. Possibly there are other potential mediation processes, such as “knowing whom” and “knowing why” career competencies that emphasize social capital and working motivations (Eby et al., 2003). Future studies may control relevant variables to substantiate such conclusions.

## Data availability statement

The data analyzed in this study is subject to the following licenses/restrictions: The data was private and not available to the public. Requests to access these datasets should be directed to yuk@ruc.edu.cn.

## Ethics statement

Ethical review and approval was not required for the study on human participants in accordance with the local legislation and institutional requirements. The patients/participants provided their written informed consent to participate in this study.

## Author contributions

RX: conceptualization, data analysis, and drafting. KY: conceptualization, data collection, and revising the manuscript. YG and PC: drafting and validating the final submitted version. All authors contributed to the article and approved the submitted version.

## Funding

This article was funded by the National Natural Science Foundation of China (No. 72171227).

## Conflict of interest

The authors declare that the research was conducted in the absence of any commercial or financial relationships that could be construed as a potential conflict of interest.

## Publisher’s note

All claims expressed in this article are solely those of the authors and do not necessarily represent those of their affiliated organizations, or those of the publisher, the editors and the reviewers. Any product that may be evaluated in this article, or claim that may be made by its manufacturer, is not guaranteed or endorsed by the publisher.
